# Pleural fluid agitation for improving the microbiologic diagnostic yield in pleural infection: a feasibility study

**DOI:** 10.1186/s12931-025-03208-7

**Published:** 2025-04-18

**Authors:** Ahmed Sadaka, Reda Said, Heba Ashmawy, Hadir Okasha, Heba Gharraf

**Affiliations:** 1 Department of Chest Diseases, Alexandria University Faculty of Medicine, Alexandria (Egypt), El- Khartoum Square, Alexandria, 21526 Egypt; 2 Department of Medical Microbiology and Immunology, Alexandria University Faculty of Medicine, Alexandria, Egypt

**Keywords:** Pleura, Pleural infection, Thoracentesis, Microbiologic yield, Thoracic ultrasound, Fluid agitation

## Abstract

**Background:**

Pleural infection is a commonly encountered respiratory disease but in > 40% the underlying microbiologic etiology is unknown. This feasibility study aims to investigate whether pleural fluid agitation prior to sample aspiration is safe and can improve the diagnostic yield of microbiologic analysis.

**Methods:**

Thirty adult patients with pleural infection, based on clinical, imaging and biochemical evidence, were included in this feasibility study. Ultrasound guided thoracentesis was performed with an initial standard aspiration sampling technique, followed by pleural fluid agitation into the pleural cavity for 3–5 cycles before collecting the agitated fluid. Coded samples were sent for biochemical and microbiologic analysis with culture in aerobic and anaerobic media.

**Results:**

No complications were encountered with the pleural fluid agitation technique. Overall, 14 (46.6%) of patients had a positive pleural fluid culture. No yield discordance was noted between the standard and the agitated pleural fluid sampling techniques except for 1 extra agitated sample growing klebsiella pneumoniae and another agitated sample with mixed infection showing an additional anaerobic bacterial growth. Four (30.8%) of the 13 concordantly positive samples showed heavier bacterial growth in the agitated samples using semi-quantitative culture scoring.

**Conclusion:**

Pleural fluid agitation was safe but didn’t significantly add to the microbiologic yield in pleural infection. However, higher bacterial growth in almost one third of positive samples suggests a potential effect for further investigation in a larger study.

**Summary at a glance:**

Despite being safe, pleural fluid agitation resulted in no significant improvement in the microbiologic yield among pleural infection. However, agitated samples grew more bacteria in almost a third of the positive samples suggesting a signal for further investigation in a larger study.

**Study registration:**

Clinicaltrials.gov - NCT05702580, 23/12/2022.

## Introduction

Pleural infection is among one of the commonly encountered respiratory diseases with significant toll on patients and the healthcare delivery system [1–3]. The rising incidence probably corresponds with the demographic changes towards a more ageing population with increasing comorbidities [4]. The associated mortality is relatively high especially among those with more risk factors [1, 4–6].

Mostly pleural infection patients have a subacute presentation with symptoms including fever, chest pain, cough and sometimes sputum that is related to an underlying pneumonia [7]. However, It has recently been shown that a considerable proportion of these patients lack radiological evidence of pneumonia. Additionally, the common finding of oropharyngeal flora among culture-positive patients supports a significant role of hematogenous spread among the different routes of seedling of the pleura [7–10]. Being predominantly polymicrobial in nature with quite a high prevalence of anaerobic bacteria, the aspirated pleural fluid should be gram stained and cultured in both aerobic and anaerobic media [8, 11]. Despite this, the microbiologic diagnostic yield remains relatively poor at 20–60%. Menzies et al. have shown that bedside pleural fluid sample inoculation in blood culture bottles could increase the yield by around 20%. [12] In another attempt to enhance sampling efficacy, the AUDIO feasibility study demonstrated that ultrasound-guided pleural biopsies could improve the diagnostic yield by 25% compared to results achieved from pleural fluid and blood cultures, independent of the presence of pleural thickening [13]. Despite the encouraging latter outcomes, not only do they need validation in a large prospective trial but this also entails more time and resource allocation while being limited to centres with experience in pleural disease management. Notably, these results suggest that the bacteria being diagnostically targeted might more likely be residing on the pleural surface with a better blood supply rather than being planktonic in the acidic, glucose deficient pleural fluid.

We thus hypothesize that an agitation of the pleural fluid prior to sample aspiration would aid in achieving a better cellular representation of the pleural space. This study aimed to investigate the safety and feasibility of an increased microbiological yield for infected pleural fluid via a pre-aspiration agitation of the pleural fluid which would have a positive effect on management and eventual patient outcomes.

## Methods

Over a 12-months period, this single centre pilot study prospectively recruited patients referred to the chest clinic or presenting to the emergency department of Alexandria main university hospital. The study was conducted in accordance with the revised declaration of Helsinki, with the protocol approved by the Ethics Committee at Alexandria University Faculty of Medicine and registered at Clinicaltrials.gov: NCT05702580, 23/12/2022.

The study included 30 adult patients with pleural infection based on clinical presentation, imaging, laboratory investigations and pleural fluid examination showing glucose < 40 mg/dL or pH < 7.2 with lower respiratory infection or pus on aspiration, in the presence of at least a moderate amount of pleural fluid collection defined as fluid spanning at least 2 intercostal spaces on thoracic ultrasound (TUS). Patients with minimal - mild pleural effusion, hemodynamic instability, uncorrected coagulopathy, transudative or exudative lymphocytic pleural effusion on biochemical analysis were excluded. The primary outcome was the difference in the diagnostic yield via microbiologic analysis while the secondary outcome measures were differences in pleural fluid biochemical parameters including protein, glucose and LDH levels.

After obtaining written informed consent forms, eligible patients underwent TUS to confirm, quantify and characterize the pleural effusion in terms of echogenicity, presence of swirling and septations and the most appropriate site for thoracentesis. A depth of at least 3 cm between the visceral and parietal pleura in absence of intervening intercostal vessels was considered safe for aspiration and agitation. Using a strict aseptic technique, a 16-18G catheter was introduced into the pleural space under local anaesthesia with aspiration of 30 ml of fluid (10 and 20 ml for standard biochemical and microbiological analysis respectively) using the standard thoracentesis procedure. For the agitation technique, a 20-ml syringe was used, with the catheter in the pleural space, to aspirate and rapidly flush the fluid back into the pleural space for 3–5 consecutive cycles before another 30 ml of agitated fluid was finally drawn into the collection syringe. Samples collected for microbiological examination, using either sampling techniques, were promptly inoculated in aerobic and anaerobic blood culture bottles before being coded and sent to the blinded local in-hospital microbiology lab. Samples were processed and subjected to standard Gram staining, direct culture on standard aerobic and anaerobic microbiological media. The results were evaluated after 48 h and 72 h and time to positivity was recorded when growth was noted. Similar coded samples were subjected to biochemical analysis.

### Statistical analysis

Power and data analyses were conducted using IBM SPSS^®^ for Windows software version 27. A calculated minimum sample size of 29 patients was needed to detect a 25% increase in microbiologic yield with a power of 80% and a 2-sided significance level of 5% using McNemar’s test. Descriptive statistics are presented as counts and percentages for categorical variables, while continuous variables are presented as mean and standard deviation for normally distributed data and median with interquartile range for non-normally distributed data. The Shapiro–Wilk test was used to test for data normality. Paired sample analysis of the different parameters from 30 participants was conducted using McNemar’s test and paired sample T-test. Spearman’s and rank-biserial correlation tests were used where appropriate. A p-value ≤ 0.05 was considered statistically significant.

## Results

Between January and December 2023, 40 patients were screened for eligibility of which 10 were excluded for not matching the eligibility criteria (5), lack of consent (3) and sample label error (2). Thirty patients completed the study with a mean age of 50.4(16.8) years, 80% of whom were males, with 22(73.3%) reporting a history of smoking (Table 1).

**Table 1:** Characteristics of the study population. Data presented as mean (SD) or number (%)

	*n* = 30
Age, years	50.4(16.8)
Males, n (%)	24(80%)
BMI, Kg.m^2^	25.8 (4.9)
Ever smokers	22 (73.3%)
RAPID score	2.7 (1.6)
≥ 1 Comorbidity*	27 (90%)
Pleural fluid	
Glucose (mg/dL)	68.4 (98.6)
Protein (g/dL)	4.5 (2.2)
LDH (U/L)	7099 (8999)
WBC count (cells/µL)	5131 (5733)

* Dental problems, drug abuse, Diabetes mellitus, respiratory/renal/heart/liver diseases, immunosuppressive states, active malignancy

Computed tomography (CT) scans of the chest revealed that 28 patients exhibited unilateral effusion, with 60% (18 patients) presenting with encysted effusions. Thoracic ultrasound exams showed that 50% (15 patients) did not have septations while 13 had simple septations, and 2 exhibited complex septations. The average fluid depth measured was 8.11(2.6) cm.

Laboratory analyses indicated an average white blood cell count of 14.1(5.2) x 10³/µL with a predominance of neutrophils, and a mean C-reactive protein level of 116.2(69.2) mg/dL. Biochemical analysis of pleural fluid showed a mean protein concentration of 4.5(2.2) g/dL, glucose concentration of 68.4(98.6) mg/dL, lactate dehydrogenase (LDH) level of 7099(8999) U/L, and white blood cell count of 5131(5733) cells/µL.

The technique was found to be safe and well-tolerated, with no complications during or after the procedure including pneumothorax as confirmed by TUS and chest X-ray. Only 1 patient reported discomfort, being present during both aspiration techniques. No adverse events were reported during the study.

**Fig. 1:**
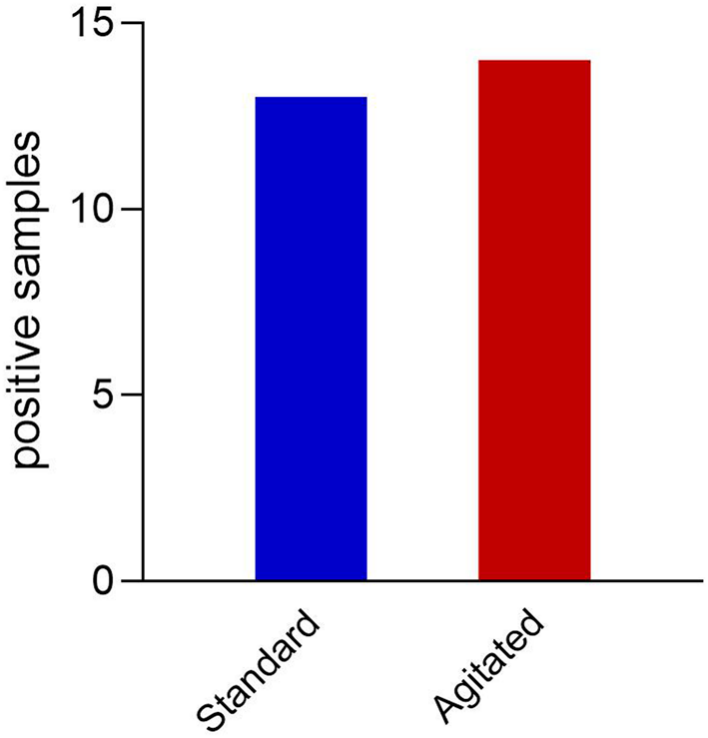
Pleural fluid culture yield after both techniques

**Fig. 2:**
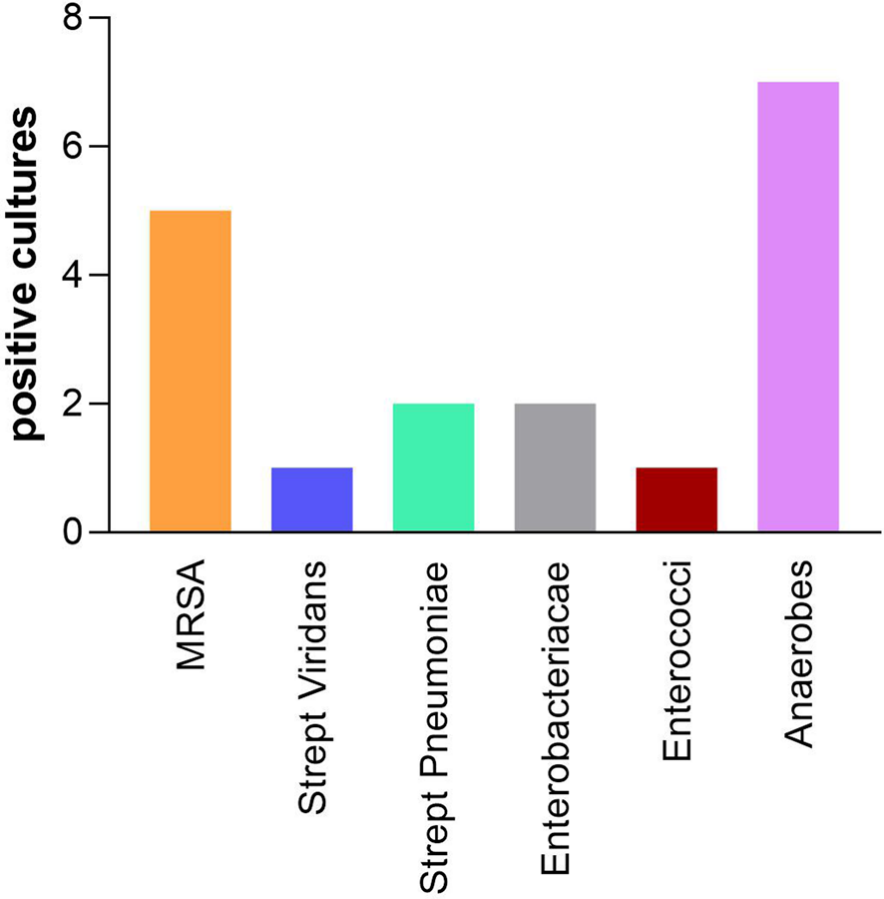
Different organisms detected in positive pleural fluid cultures

About 46.6% of patients had a positive pleural fluid culture result, indicating the presence of bacterial infection. When comparing the agitated pleural fluid sampling technique to standard methods, there was generally no significant discordance in yield as depicted in Figure 1. However, there were two exceptions: one case where the agitated sample identified Klebsiella pneumoniae not found in the standard sample, and another where the agitated sample detected an additional anaerobic bacterial growth. No correlation was found between different sonographic or biochemical characteristics of pleural fluid and culture positivity or time to culture positivity. The organisms detected among positive pleural fluid samples are demonstrated in Figure 2.

Among those with concordant positive pleural cultures, 30.8% (4/13) showed evidence of higher bacterial growth in the agitated samples compared to the standard samples when assessed using semi-quantitative scoring methods. Regarding the biochemical analysis of pleural fluid, the average protein levels were 4.68 g/dL and 4.53 g/dL, lactate dehydrogenase (LDH) levels were 7099 U/L and 7376.7 U/L, and glucose levels were 68.4 mg/dL and 75.4 mg/dL in the standard and agitated samples, respectively.

## Discussion

This feasibility study investigating a novel sampling technique among patients with pleural infection showed no evidence of significant improvement in microbiologic yield. Despite the apparently negative results, the agitated sampling technique results were not identical to the standard method with few samples showing positivity or revealing more organisms or evidence of higher bacterial load.

The current microbiologic yield offered by the standard culture-based methods among patients with pleural infection is suboptimal. Even with the combined usage of standard and blood culture media for pleural fluid samples, at least 40% of patients lack a microbiologic diagnosis [8]. This reflects on higher antibiotic utilization with more economic burden and risk of adverse effects to patients. The AUDIO study has shown higher yield via ultrasound-guided pleural biopsy suggesting residence of more bacterial cells on the pleural surface compared to the pleural fluid [13]. Correspondingly, a higher diagnostic yield is known to be achieved in tuberculous effusions via the use of pleural biopsies rather than fluid samples [14]. However, this procedure needs specialist experience and is associated with more cost and time for sample acquisition. Agitation of the pleural fluid was hypothesized to: (a) improve the representativeness of the sample; (b) dislodge more bacterial cells off the pleural surface into the aspirated pleural fluid. Mohamed et al. have demonstrated that the diagnostic yield of pleural lavage, in the setting of thoracoscopy for undiagnosed exudative pleural effusions, is superior to that from conventional pleural fluid samples and can enhance that from pleural biopsies [15]. Whether fluid agitation can offer an increased diagnostic sensitivity in those with suspected tuberculous or malignant effusions is unknown and planned to be investigated.

The eligibility criteria for the study were unrestrictive, mainly excluding the inclusion of those with small amount of pleural fluid for safety purposes. Most of the participants (90%) had at least one comorbidity/risk factor for pleural infection with a median duration of symptoms of 20 days before hospital presentation which is a bit longer than that reported by Maskel et al. [16] A more delayed presentation is known to be associated with worse outcomes but whether this impacts microbiologic results from pleural fluid samples is unclear, especially as infection transitions from the fibrinopurulent into the organizing stage [17]. 

The agitation sampling technique was not associated with any complication or relevant pain. It was though notable that both aspiration techniques were mostly resemblant in terms of results. Analyzing different characteristics of patients or pleural fluid pertaining to severity did not show any predictor of gram stain/culture positivity. No difference in microbiologic yield was noted among those having frank pus on aspiration. Similarly, pleural septations showed no correlation with positive culture results contrary to findings reported by Barnes et al. [18] Regression analysis did not identify any patient characteristic or fluid character that is associated with increased likelihood of bacterial growth. This can be attributed to the limited number of positive cultures.

It is worth highlighting that 4(28.5%) of the agitated positive samples revealed higher colony counts using semiquantitative scoring method, compared to the growth in the standard samples. Semiquantitative culture use in respiratory infections offers good diagnostic accuracy and correlation with quantitative techniques while being a relatively quick, easy and inexpensive method for bacterial isolation [19, 20]. This finding suggests a potentially higher viable bacterial load in samples aspirated after pleural fluid agitation. It is notable however that despite the different semi-quantitative results, the time to culture positivity was similar across both techniques and thus didn’t lead to a significant or earlier change to antibiotic regimens prescribed. Additionally, relative discordance was noted where klebsiella and anaerobic growth were demonstrated in 2 agitated samples but not in their paired standard ones. That former klebsiella growth warranted a switch to targeted antibiotic therapy in a single patient.

### Critique of the method

We based our sample size on the assumption of a 25% difference in microbiologic yield, yet the study might have been under-powered for detection of smaller yet clinically significant differences between both sampling techniques. Additionally, the studied participants from this single center are relatively younger compared to usually reported pleural infection demographics. Higher sensitivity diagnostic molecular techniques such as 16s-ribosomal RNA testing were not used, given the aim was to investigate easily feasible and cost-effective methods to increase diagnostic accuracy. The number of agitation cycles was set at 3–5 as per protocol regardless of the fluid amount or loculation but it was not investigated whether more agitation cycles could contribute to different results. Pleural infections of various degrees of severity were included with no adverse events recorded. This allowed proper representation of the different RAPID proportions like those reported in MIST1 and MIST2 cohorts but with much lower numbers included especially in the low and medium risk categories (5 and 10 patients respectively) which would limit the results conclusiveness in this population. A strength of the study, however, is having paired samples from each patient with attention to the sampling sequence to maintain validity of the results as well as the use of blood culture media to improve the diagnostic yield. The concealment of the aspiration method was maintained in the provided samples for ensuring blinding of laboratory personnel to limit detection bias.

## Conclusion

Despite this feasibility study showing lack of significant improvement in microbiologic yield with the agitated pleural fluid sampling technique, it was found to be safe. Evidence of higher microbial load in the agitated samples can suggest a potential benefit that needs to be investigated in a larger cohort with different number of agitation cycles. This might have important implications not only for increasing microbial yield but further to guide faster and more specific adjustment of prescribed antibiotic regimens, aid outcome prediction as well as reduce the pressure drive for antimicrobial resistance.

## Data Availability

No datasets were generated or analysed during the current study.
